# OCT4 induces embryonic pluripotency via STAT3 signaling and metabolic mechanisms

**DOI:** 10.1073/pnas.2008890118

**Published:** 2021-01-15

**Authors:** Giuliano G. Stirparo, Agata Kurowski, Ayaka Yanagida, Lawrence E. Bates, Stanley E. Strawbridge, Siarhei Hladkou, Hannah T. Stuart, Thorsten E. Boroviak, Jose C. R. Silva, Jennifer Nichols

**Affiliations:** ^a^Wellcome Trust—Medical Research Council Stem Cell Institute, Jeffrey Cheah Biomedical Centre, University of Cambridge, CB2 0AW Cambridge, United Kingdom;; ^b^Living Systems Institute, University of Exeter, EX4 4QD Exeter, United Kingdom;; ^c^Department of Pharmacological Sciences, Icahn School of Medicine at Mount Sinai, New York, NY 10029;; ^d^Department of Biochemistry, University of Cambridge, CB2 1GA Cambridge, United Kingdom;; ^e^Department of Physiology, Development and Neuroscience, University of Cambridge, CB2 3EG Cambridge, United Kingdom;; ^f^Centre for Trophoblast Research, University of Cambridge, CB2 3EG Cambridge, United Kingdom

**Keywords:** developmental biology, single-cell profiling, metabolism, STAT3 pathway, OCT4

## Abstract

OCT4 is a fundamental component of the molecular circuitry governing pluripotency in vivo and in vitro. To determine how OCT4 establishes and protects the pluripotent lineage in the embryo, we used comparative single-cell transcriptomics and quantitative immunofluorescence on control and OCT4 null blastocyst inner cell masses at two developmental stages. Surprisingly, activation of most pluripotency-associated transcription factors in the early mouse embryo occurs independently of OCT4, with the exception of the JAK/STAT signaling machinery. Concurrently, OCT4 null inner cell masses ectopically activate a subset of trophectoderm-associated genes. Inspection of metabolic pathways implicates the regulation of rate-limiting glycolytic enzymes by OCT4, consistent with a role in sustaining glycolysis. Furthermore, up-regulation of the lysosomal pathway was specifically detected in OCT4 null embryos. This finding implicates a requirement for OCT4 in the production of normal trophectoderm. Collectively, our findings uncover regulation of cellular metabolism and biophysical properties as mechanisms by which OCT4 instructs pluripotency.

## Significance

We used single-cell whole-genome transcriptional profiling and protein quantification to investigate the role of OCT4 in establishing pluripotency in the murine embryo. Surprisingly, most pluripotency-associated factors are induced normally in OCT4 null early blastocysts, apart from members of the STAT3 signaling pathway. Coincidentally, certain trophectoderm markers are induced but not *Cdx2*, which was previously implicated to repress *Pou5f1* in vitro. This ectopic gene activation suggests a role for OCT4 in maintaining chromatin in a pluripotency-compatible state, likely via UTF1, a known OCT4 target. At implantation, OCT4 null inner cell masses morphologically resemble trophectoderm but exhibit molecular differences linking metabolic and physical stress responses to loss of OCT4. These effects correlate with reduced STAT3 signaling and consequent reduction of oxidative respiration.

Formation of a mammalian organism pivots upon the establishment of extraembryonic tissues to pattern the fetus and expedite connection with the maternal vascular system while preserving a pluripotent population of cells with the responsive capacity to generate body pattern and tissues progressively during development. The specification of trophectoderm (TE, founder of the placenta) on the outside of the preimplantation embryo coincides with the appearance of the blastocyst cavity and a metabolic switch from pyruvate and lactose to glucose utilization with increased oxygen consumption (1–5). This heralds an increase in metabolic activity by the differentiating TE (6, 7). The murine embryo can overcome adverse consequences associated with accumulation of reactive oxygen species during the metabolic transition to oxidative phosphorylation, facilitated by the transcriptional enhancer factor TEAD4 (8, 9). TEAD4 intensifies in the TE, where it cooperates with nuclear YAP to initiate transcription of TE-specific genes (10, 11). Acquisition of TE identity actuates distinct metabolic requirements compared with the undifferentiated inner cell mass (ICM). During blastocyst expansion, the transcription factor OCT4 (encoded by *Pou5f1*) becomes restricted to the ICM (12). OCT4 is essential for the establishment of the pluripotent epiblast (EPI), preventing differentiation of the embryo toward TE (13) and propagation of pluripotent stem cells in vitro (13–17). Studies in embryonic stem cells (ESC) indicate that the pluripotency network hinges upon OCT4 (18–22). In the embryo, OCT4 is detected throughout cleavage (12), whereas many other pluripotency-associated factors, such as NANOG, appear after the onset of zygotic genome activation (23). However, in embryos lacking OCT4, NANOG emerges robustly (24, 25), ruling out failure to express this key pluripotency network gene as a contributing feature of the OCT4 null phenotype. To date, evidence that all cells in OCT4 null embryos adopt a TE identity is largely restricted to morphology and expression of TE-specific markers at the time of implantation (13, 24, 26). To scrutinize how acquisition of pluripotency fails in OCT4 null ICMs, we used single-cell RNA sequencing (scRNAseq) and quantitative immunofluorescence (QIF) to examine gene expression in wild-type (WT), heterozygous (HET), and OCT4 null mid- and late-blastocyst ICMs. Differences between samples and groups, calculated using bioinformatics and computational analysis, revealed a role for OCT4 in defining the metabolic, pluripotent, and biophysical status of the murine ICM.

## Results

### Divergence of OCT4 Null from Control ICM Cells during Blastocyst Expansion.

**Fig. 1.**
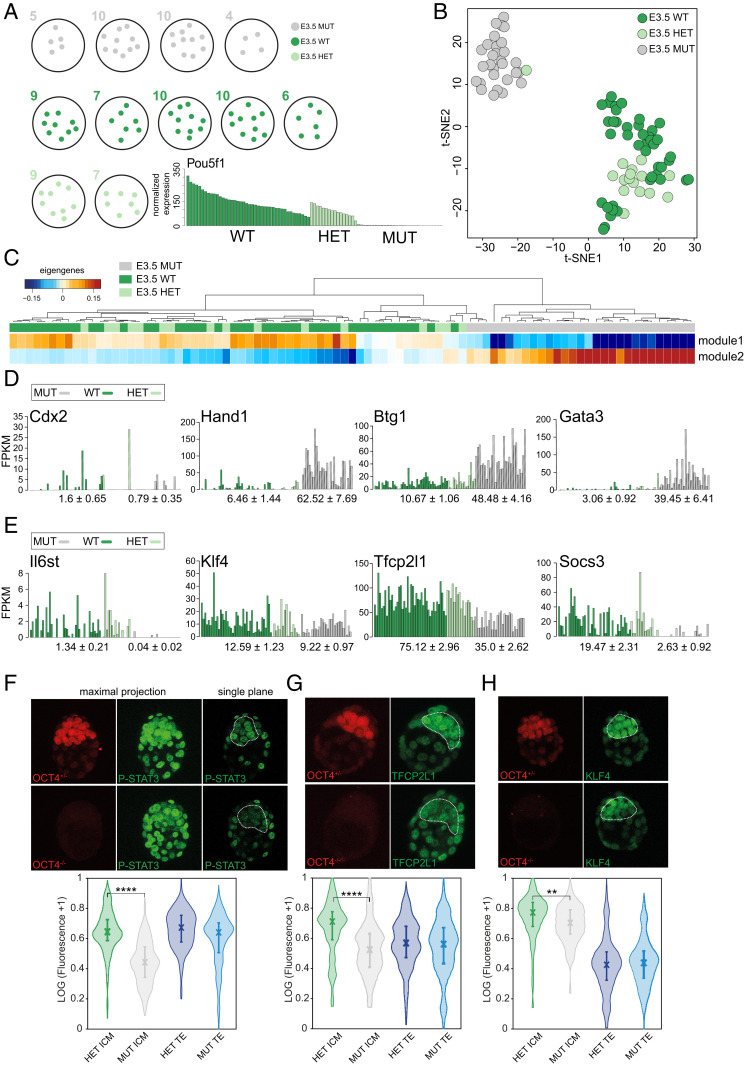
(*A*) Schematic of number of single cells per embryo (E3.5 stages) and their genotype. The bar plot shows FPKM expression of *Pou5f1* for each single cell. (*B*) t-distributed stochastic neighbor embedding plot for early blastocyst cells. The sample color represents the different genotypes. (*C*) One-way hierarchical cluster of eigengenes values (weighted average expression profile) computed from WGCNA (power 10; distance = 0.35, size = 30). (*D*) Bar plot of FPKM expression of selected TE markers and mean ± SD for WT/HET and MUT (*P* adjusted *Cdx2*: 0.96, *Hand1:* 2.32 × 10^−10^, *Btg1:* 2.32 × 10^−10^, and *Gata3:* 2.32 × 10^−10^). (*E*) FPKM expression of genes in STAT3 pathway (*P* adjusted *Il6st*: 1.35 × 10^−15^, *Klf4*: 1, *Tfcp2l1*: 5.88 × 10^−10^, and *Socs3*: 1.45 × 10^−16^). (*F*) Confocal images and normalized expression of OCT4 HET and MUT embryos stained for p-STAT3, (*G*) TFCP2L1, and (*H*) KLF4 and corresponding violin plots of quantitative immunofluorescence analysis.

To investigate the cause of ICM failure in the absence of OCT4, scRNAseq was performed. ICMs were immunosurgically isolated from embryonic day (E) 3.5 (mid) blastocysts resulting from *Pou5f1* HET inter se mating. ICMs were genotyped using TE lysate (13, 27). Quality control, as previously reported (28), eliminated inadequate samples, leaving 29 mutant (MUT), 42 WT, and 16 HET cells from four, five, and two mid-blastocysts, respectively (Fig. 1*A* and *SI Appendix*, Table S1). *Pou5f1* RNA was absent from MUT ICM cells, confirming degradation of maternal transcripts (Fig. 1*A* and *SI Appendix*, Fig. S1*A*) consistent with the lack of OCT4 protein observed at the morula stage (13). To characterize global differences and similarities between genotypes, t-distributed stochastic neighbor embedding analysis was performed (Fig. 1*B* and *SI Appendix*, Fig. S1*A*) using the most variable genes identified in E3.5 blastocysts (*n* = 2,232, log_2_FPKM [fragments per kilobase of transcript per million mapped reads] > 0.5, logCV^2^ > 0.5). MUT cells cluster separately from HET and WT, suggesting changes in transcriptome.

Weighted gene correlation network analysis (WGCNA) allows for the extraction of modules defined by coregulated genes combined with unsupervised clustering (Fig. 1*C*). Two main modules emerged: module 1 coclusters HET and WT and coregulates pluripotency-associated genes such as *Pou5f1*, *Gdf3*, and *Zfp42* (29–31); module 2 is specific for MUT cells, expressing established TE markers, including *Gata3*, *Hand1*, and *Krt18* (32–35) (*SI Appendix*, Fig. S1*B* and Table S2). Interestingly, HET and WT cells clustered together, indicating no more than a negligible effect of reduced *Pou5f1* in HET embryos, contrasting with the elevated and more homogeneous expression of *Nanog*, *Klf4*, and *Esrrb* previously reported in *Pou5f1* HET ESCs (36) (*SI Appendix*, Fig. S1*C*).

### Suppression of TE Gene Network in the ICM Depends on OCT4.

In light of the significant transcriptional differences revealed above, we sought insight into regulation of pluripotency genes in E3.5 WT/HET and MUT ICM cells. Consistent with previously published immunohistochemistry (IHC) (24, 25), *Nanog* was detected, albeit heterogeneously, in MUT cells (*SI Appendix*, Fig. S1*D*). Conversely, *Sox2* was not significantly affected at either RNA or protein levels, as revealed by QIF (*SI Appendix*, Fig. S1 *D* and *E*) (37). *Esrrb*, reported to be a direct OCT4 target in vivo (24), showed modest down-regulation in MUT cells by scRNAseq but no obvious difference at the protein level via QIF (*SI Appendix*, Fig. S1 *D* and *E*), suggesting initiation of expression independent of OCT4. Specific chromatin components establish and maintain pluripotency (38). *Utf1*, a direct OCT4 target (39), is expressed in normal ICM and EPI (40); its expression decreases upon differentiation (41), consistent with its role in maintaining a chromatin structure compatible with self-renewal in vitro (42). *Utf1* was not detected in MUT blastocysts (*SI Appendix*, Fig. S1*D*). TE markers, such as *Hand1*, *Gata3*, and *Btg1*, were found in most MUT cells, whereas *Cdx2* was poorly represented (5/29 MUT cells; Fig. 1*D*), suggesting that TE differentiation of MUT cells is not primarily directed by *Cdx2*, although its protein appeared in the majority of later OCT4 null ICMs by E4.0 (26).

### Reduction of JAK/STAT Signaling Distinguishes OCT4 Null ICMs.

The JAK/STAT signaling pathway is fundamental for self-renewal and pluripotency in vivo and in vitro (43–45). Active P-STAT3 protein and its targets *Klf4* (46) and *Tfcp2l1* (47) were significantly lower in MUT cells at both messenger RNA (mRNA) and protein levels (Fig. 1 *E*–*H*). Total *Stat3* mRNA did not vary (*SI Appendix*, Fig. S1*F*). Reduced STAT3 signaling in MUT embryos was most likely attributable to the absence of its upstream cytokine receptor subunit, *gp130* (*Il6st*; Fig. 1*E*), also a putative target of OCT4 in ESC (*SI Appendix*, Table S3; https://chip-atlas.org/). *Socs3*, a STAT3 target that exerts negative feedback regulation (48), was barely detectable in MUT cells (Fig. 1*E*). Principal component analysis (PCA) computed with JAK/STAT signaling pathway genes (https://www.genome.jp/kegg/) segregates MUT from WT/HET cells (*SI Appendix*, Fig. S1*G*); the cumulative sum on the relative percentage of gene expression is significantly higher (*P* < 0.05) in WT/HET, indicating down-regulation of this pathway in MUT cells (*SI Appendix*, Fig. S1*H*). Consistent with a role for OCT4 in control of STAT3 signaling, we observed a rapid increase in pSTAT3 following overexpression of OCT4 in ESCs (*SI Appendix*, Fig. S1 *I* and *J*).

### Dissecting Overt Impairment of Lineage Segregation in Mature OCT4 Null ICMs.

**Fig. 2.**
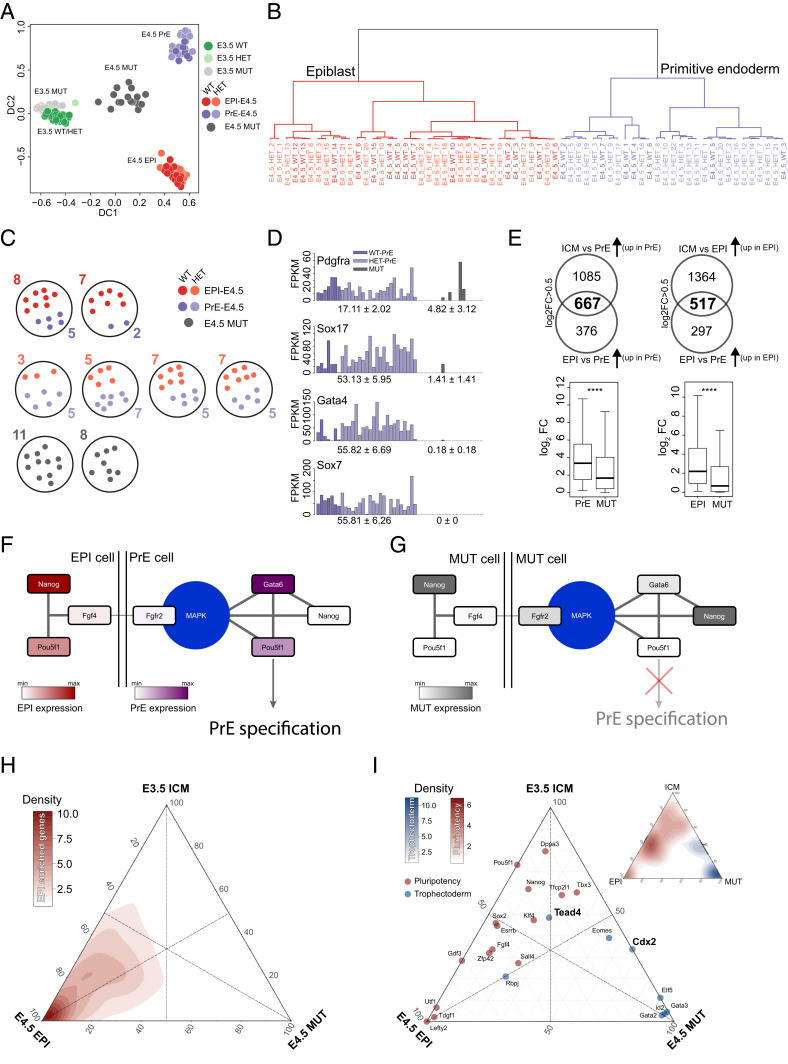
(*A*) Diffusion plot of early and late blastocyst cells; color represents the different genotypes and lineages. (*B*) Dendrogram (agglomeration method: ward.D2) for late blastocyst ICM WT/HET cells and (*C*) schematic representation of the number of late ICM single cells per embryo and their genotype. (*D*) Single-cell FPKM expression of PrE markers and mean ± SD (*P* adjusted *Pdgfra*: 0.11, *Sox17*: 2.75 × 10^−10^, *Gata4*: 1.62 × 10^−10^, and *Sox7*: 1.62 × 10^−10^) in PrE and MUT cells. (*E*, *Top*) Venn diagram showing the number of significant (*P* adjusted < 0.05) and enriched PrE and EPI genes. (*E*, *Bottom*) Box plot of log_2_FPKM in late blastocyst PrE and MUT cells of 667 genes and late blastocyst EPI and MUT cells of 517 genes. (*F*) Network of genes associated with PrE specification in WT cells and (*G*) MUT cells. (*H*) Ternary plot of early WT/HET early blastocyst cells, WT/HET EPI, and MUT cells. Axes show the relative fraction of expression of 814 EPI-enriched genes or (*I*) pluripotent and TE-associated genes.

The results so far reveal a reduced expression of direct OCT4 targets and JAK/STAT pathway members in MUT ICMs coincident with the ectopic activation of selected TE genes, indicating transcriptional divergence in MUT cells by E3.5. For a detailed characterization of the diversion of ICM toward TE in embryos lacking OCT4, diffusion component analysis was performed on ICMs isolated immunosurgically from implanting embryos at E4.5 (Fig. 2*A* and *SI Appendix*, Fig. S2*A*); 19 cells isolated from 2 MUT, 22 from 2 WT, and 44 from 4 HET E4.5 ICMs were analyzed (*SI Appendix*, Table S1 and Fig. 2 *B* and *C*). The expression level of *Pou5f1* was measured in each cell (*SI Appendix*, Fig. S2*B*). WT and HET cells assume identity of either EPI or primitive endoderm (PrE): 37 versus 29, respectively (Fig. 2 *A*–*C* and *SI Appendix*, Fig.S2 *A* and *B*). No E4.5 MUT cells cluster near EPI or PrE (Fig. 2*A* and *SI Appendix*, Fig. S2*A*). ScRNAseq failed to identify significant expression of maturing PrE markers such as *Sox17*, *Gata4*, or *Sox7* (Fig. 2*D*) in E4.5 MUTs, as predicted from IHC or bulk RNA analysis (24, 25). Rarely, E4.5 MUT cells expressed *Pdgfrα* (Fig. 2*D*), probably reflecting initiation of expression prior to loss of maternal OCT4 since PDGFRα, like GATA6, is an early presumptive PrE marker (49, 50).

WGCNA revealed independent clustering of MUT cells and coexpression of specific genes normally mutually exclusive by E4.5 (*SI Appendix*, Fig. S2*C* and Table S4). We assessed quantitatively and qualitatively the PrE and EPI genes underrepresented in E4.5 MUTs (Fig. 2*E* and *SI Appendix*, Fig. S2*D*) and observed a significant drop in intensity in MUT cells, suggesting global failure to activate both PrE and EPI transcription networks. In normal late blastocysts, *Gata6* becomes restricted to a subset of cells constituting the PrE. As expected, in WT/HET embryos, its expression is mutually exclusive with *Nanog* (50, 51). However, in E4.5 MUTs, 7/19 cells coexpressed *Gata6* and *Nanog* (*SI Appendix*, Fig. S2*E*), confirming a role for OCT4 in mediating mutual repression (24). PrE induction and differentiation is induced by FGF4 produced from EPI cells (52) interacting with FGFR1 and FGFR2 (53–55). The failure of this early lineage segregation in E4.5 MUT ICMs confirms the requirement for OCT4 induction of FGF4 (13); consequently, E4.5 MUT cells express only minimal *Fgf4* but up-regulate *Fgfr1* and *Fgfr2* (*SI Appendix*, Fig. S2*F*). We adapted a model of the gene network directing the second lineage decision, EPI versus PrE (56), in WT/HETs compared with MUT cells. In the presence of OCT4, EPI cells express NANOG and FGF4 (Fig. 2*F*). FGF4 drives PrE fate transition and restriction (57) by triggering ERK signaling, suppressing NANOG, and activating PrE markers SOX17, GATA4, and SOX7. However, in E4.5 MUT cells, the ERK signal is disrupted and generally down-regulated (*SI Appendix*, Fig. S2*G*), resulting in the absence of PrE markers (Fig. 2*G*).

Having identified normal expression of some pluripotency factors in mid-MUT embryos, we inspected late blastocyst ICMs for EPI-enriched genes (*n* = 814, Fig. 2*E*). Ternary plots represent the expression density between three different conditions. We reasoned that if MUT cells fail to express EPI-enriched genes globally, a bias in the density distribution would be expected. Indeed, the EPI/ICM sides of the triangle showed the highest density for EPI-enriched genes when compared with MUT (Fig. 2*H*). We then explored the distribution of pluripotency and TE-associated factors along the ternary plot. Genes not expressed in MUT cells localize close to the EPI apex; these include *Utf1*, *Lefty2*, and *Tdgf1*. Overall, most pluripotency factors cluster at the ICM/EPI side, indicating lower expression in the E4.5 MUT cells (Fig. 2*I*) or TE cells (*SI Appendix*, Fig. S2*H*). Conversely, genes associated with TE identity, *Gata2*, *Gata3*, *Eomes*, *Id2*, *Elf5*, and the Notch signaling pathway (35, 58–62), localize on the side specific for MUT (Fig. 2*I*) and TE cells (*SI Appendix*, Fig. S2*H*). Interestingly *Tead4*, a crucial transcriptional regulator of mitochondrial function in TE, is down-regulated in MUT cells, suggesting impairment of mitochondrial function uncoupled from the apparent TE identity of E4.5 MUT ICM cells (Fig. 2*I*).

### OCT4 MUT Cells Acquire TE-like Identity but Diverge from Normal TE.

**Fig. 3.**
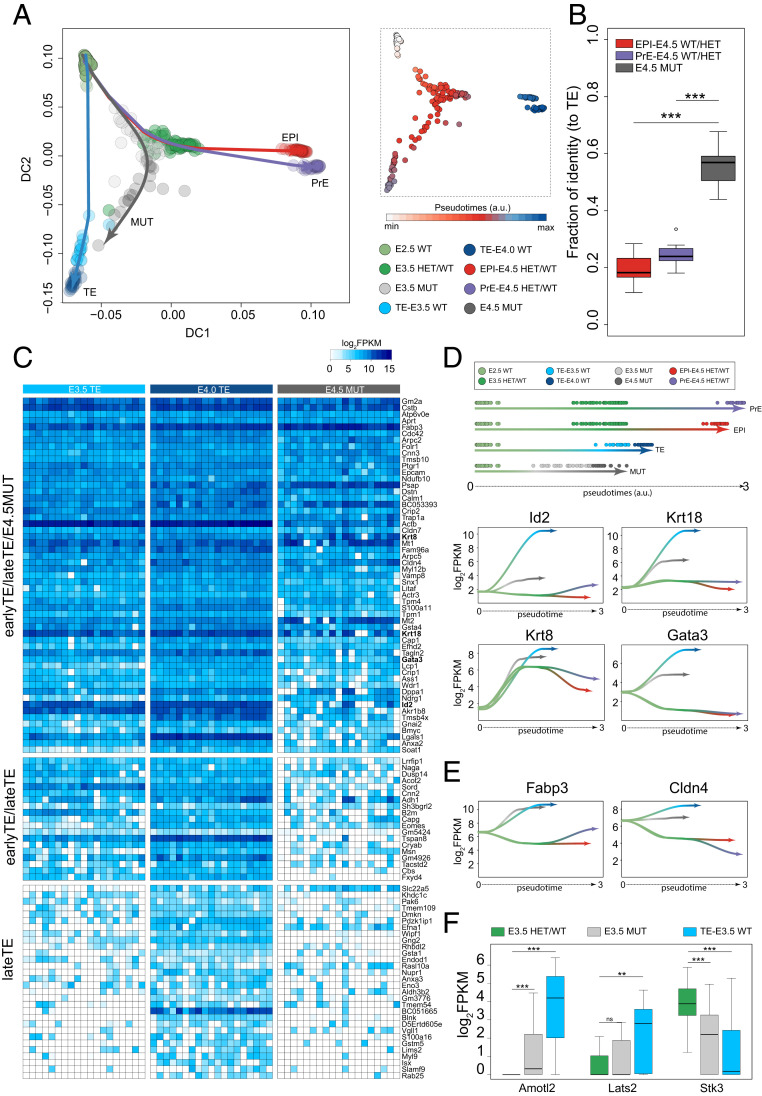
(*A*, *Left*) Diffusion component plot for E3.5/E4.5 WT/HET/MUT cells (this study) and E3.5 and E4.0 TE cells from ref. 63. Color represents the different genotypes/lineages. Trajectory lines were fitted with cubic line (lambda = 0.01). (*A*, *Right*) Diffusion component and pseudotime expression. (*B*) Fraction of similarities between E4.5 EPI (WT/HET)/E4.5 PrE (WT/HET)/E4.5 MUT and E4.0 TE cells computed using all expressed genes (log_2_FPKM > 0; Student’s *t* test, ***P < 0.001). (*C*) Heat map of TE markers identified by ref. 92 between ICM and TE single cells. (*D*) Identification of lineage trajectories and loess curve fitting between pseudotimes and log_2_FPKM for *Id2*, *Krt18*, *Krt8*, and *Gata3*. (*E*) Loess curve fitting between pseudotimes and log_2_FPKM for *Fabp3* and *Cldn4*. (*F*) Box plot of FPKM expression of genes in Hippo signaling pathway (Student’s *t* test; *P < 0.05, **P < 0.01, ***P < 0.001).

To understand how OCT4 represses TE transcription factors during normal ICM development, we sought to identify exclusive and common gene expression between WT TE and E4.5 MUT ICM cells. We consulted published TE single-cell data from E3.5 and E4.0 embryos (63). TE from our own samples was not included because by E4.5, embryos have undergone mural TE giant cell transformation and are therefore technically impossible to disaggregate without destroying RNA quality. Diffusion component analysis, coupled with pseudotime reconstruction and nonlinear regression, identified different developmental trajectories (Fig. 3*A*). The loss of OCT4 and subsequent activation of TE genes drives E4.5 MUT cells toward WT TE. Deconvolution of heterogeneous populations (64) is designed to estimate percentage identity of distinct cells toward a specific endpoint. To quantify similarities between published E4.0 TE and our E4.5 EPI/PrE (WT/HET)-E4.5 MUT cells, we computed the fraction of identity. The similarity between TE and MUT cells was the highest with a median value of ∼0.6 (60%), compared to ∼0.2 (20%) and ∼0.25 (25%) with EPI and PrE cells, respectively (Fig. 3*B*). We further validated this result with Gene Set Enrichment Analysis by comparing the rank of differentially expressed genes between E4.5 EPI (PrE)/E4.0 TE and E4.5 EPI (PrE)/E4.5 MUT (*SI Appendix*, Fig. S3 *A* and *B*). These results indicate that late blastocyst MUT cells share a significant portion of the TE transcriptional program. Since our embryos were dissected from nascent implantation sites, they are more advanced than those exhibiting non-TE identity profiled in bulk RNA-seq previously (24). We performed a two-way hierarchical analysis with published TE-enriched genes (63) (Fig. 3*C*). Transcripts enriched in early and late TE cells, such as *Id2*, *Krt18*, *Krt8*, and *Gata3* (34, 61, 65, 66), were also up-regulated in MUT cells (Fig. 3*D*). Interestingly, we also detected expression of *Fabp3* and *Cldn4* in E4.5 MUT ICM cells and confirmed this observation using OCT4 depleted ESC (Fig. 3*E* and *SI Appendix*, Fig. S3*C*). *Fabp3* regulates fatty acid transport in trophoblast cells and plays a central role in fetal development (67). *Cldn4* is essential for tight junction formation between TE cells during blastocyst formation (68). As suggested by pseudotime and diffusion component analysis, E4.5 MUT ICM cells fail to express a proportion of late TE markers.

Hippo signaling promotes the first lineage decision in mouse embryos (10, 69). Consistent with the roles of STK3, AMOTL2, and LATS2 in the Hippo pathway, their transcripts were differentially regulated in TE versus MUT ICM cells from E3.5 blastocysts (Fig. 3*F*). *Lats2* and *Amotl2* were also significantly up-regulated in OCT4 deleted ESC compared to WT and were targets of OCT4 ChIP-seq in ESC (*SI Appendix*, Fig. S3 *D* and *E*). Moreover, together with “Signaling pathways regulating pluripotency of stem cells” and “Wnt Signaling pathway,” “Hippo signaling pathway” is among the top five significant KEGG (Kyoto Encyclopedia of Genes and Genomes) pathways enriched with the top 1,000 targets of OCT4 in ESC (*SI Appendix*, Fig. S3*F*). This suggests a potential role for OCT4 in controlling the balance of Hippo signaling to prevent ectopic differentiation to TE in the normal ICM. In the absence of OCT4, ICM cells undergo default expression of a combination of specific early TE transcription factors, signaling pathways, and metabolic genes.

### Role of OCT4 in Regulation of Metabolism.

**Fig. 4.**
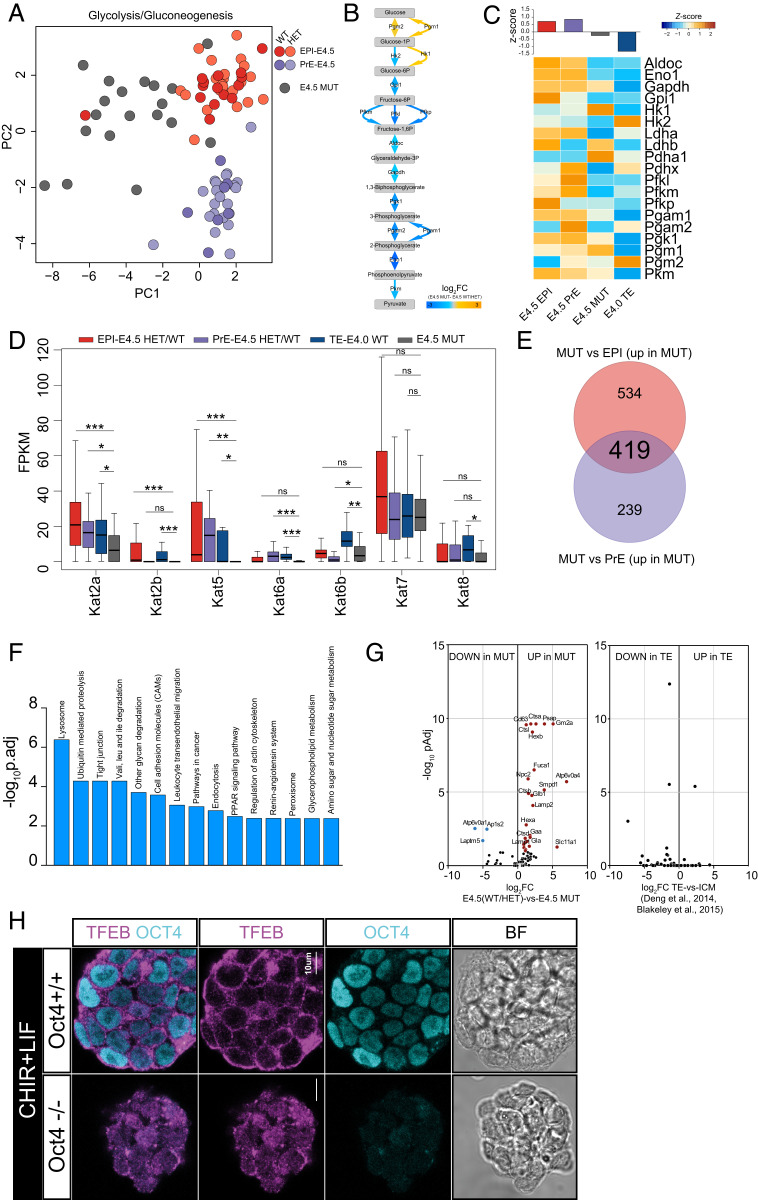
(*A*) PCA plot of E4.5 WT/HET and MUT cells computed with genes in glycolysis/gluconeogenesis KEGG pathway. (*B*) Glycolysis pathway with the associated enzymes (arrows) colored by the ratio between E4.5 WT/HET and MUT cells and (*C*) heat map of the associated enzymes. (*D*) Box plot of Kats gene expression value in E4.5 EPI/PrE WT/HET, E4.0 TE, and E4.5 MUT (Student’s *t* test; *P < 0.05, **P < 0.01, ***P < 0.001). (*E*) Number of variable genes between E4.5 WT EPI/MUT and E4.5 WT PrE/MUT. (*F*) Enrichment of KEGG pathways computed with 419 common variable genes between MUT and E4.5 EPI/PrE. (*G*) Volcano plot of lysosomal genes variable between E4.5 WT/HET and E4.5 MUT and between WT TE and WT ICM. (*H*) Confocal analysis of TFEB localization in OCT4^+/+^ and OCT4^−/−^ ESCs cultured in CHIR + LIF.

It was previously suggested that OCT4 null embryos exhibit defective metabolism by the mid to late blastocyst stage (24) and that changes in acetyl-CoA, mediated by glycolysis, control early differentiation (70). We performed PCA with glycolytic genes. Dimension one, which explains the largest variability, segregates MUT from EPI/PrE cells (Fig. 4*A*). The majority of enzymes were down-regulated in MUT cells (Fig. 4 *B* and *C* and *SI Appendix*, Fig. S4*A*). Interestingly, the rate-limiting glycolytic enzymes *Hk2* and *Pkm* together with *Eno1* and *Pgk1* are potential targets of OCT4 (*SI Appendix*, Fig. S4*B* and Table S3) in ESC. Interestingly, we observed a consistent and significant down-regulation of several KATs enzymes (Fig. 4*D*), which rely on acetyl-CoA, a product of glycolysis, to maintain the open chromatin structure associated with pluripotency. This suggests that OCT4 indirectly provides sufficient acetyl-CoA to support an open chromatin state (71). These observations are consistent with recent analysis (72) showing that OCT4 is critical to maintain a permissive chromatin environment.

To systematically assess the modulated biological processes and pathways, we identified 419 common variable genes between E4.5 MUT/E4.5 EPI and E4.5 MUT/E4.5 PrE (Fig. 4*E*) and computed KEGG pathway enrichment (Fig. 4*F*). “Tight junction,” “Cell adhesion molecule,” and “Regulation of actin cytoskeleton” processes suggest that OCT4 regulates important components of biophysical properties of ICM cells. Interestingly, the most significant enriched process was “Lysosome,” indicating a strong and pivotal role of this pathway in MUT cells. “Lysosome,” “Autophagy,” and “Tight junction” were also among the KEGG pathways enriched between WT and OCT4 deleted ESC (*SI Appendix*, Fig. S4*C*) (19). Finally, processes related to “Lysosome” were also significantly enriched, including “Peroxisome,” “Glycerophospholipid Metabolism,” “Endocytosis,” “PPAR signaling pathway,” and “Valine, leucine, and isoleucine degradation.” The most significant biological processes associated with the OCT4 MUT phenotype in late blastocysts and OCT4 deleted ESCs therefore implicate metabolism and biophysical properties.

### Members of the Lysosomal Pathway Are Specifically Activated in MUT Cells.

To determine whether activation of the lysosomal pathway is a TE characteristic, we explored differentially expressed genes and found that MUT cells, but not WT TE, up-regulated a significant proportion of lysosomal genes (Fig. 4*G*). A lysosome is essential for recycling, recruitment of lipids via autophagy and hydrolases, and for redistribution of catabolites to maintain cellular function (73). Autophagy is a catabolic response to starvation (74). Most autophagy-related genes, such as *Atg*, were up-regulated in MUT cells and OCT4 conditionally deleted ESC (Tables 1 and 2 and *SI Appendix*, Fig. S4 *D*–*F*). Moreover, MUT cells undergo a significant up-regulation of fatty acid degradation genes (*SI Appendix*, Fig. S4*E*). Our results therefore indicate that, in response to an altered and energy-insufficient metabolism, MUT cells up-regulate lysosomal and autophagy pathways as a means to provide cellular energy. The master regulator of lysosomal biogenesis and autophagy is TFEB (74). TFEB is dissociated by inactive mTORC1 and migrates into the nucleus to activate lysosomal/autophagy genes. The positive regulator of mTORC1 (*Rptor*) is down-regulated in MUT cells and, consistently, we found up-regulation of *Deptor*, a known negative regulator of mTORC1 (75) (*SI Appendix*, Table S5). To confirm activation of the lysosomal pathway via TFEB, we performed IHC on OCT4 conditionally depleted ESCs. In OCT4-positive cells, TFEB is localized mainly in the cytoplasm. After OCT4 deletion, a significant translocation of TFEB from the cytosol to the nucleus occurs (Fig. 4*H* and *SI Appendix*, Fig. S4*G*). Together, these results indicate that in response to an altered and energy-insufficient metabolism, MUT cells up-regulate lysosomal and autophagy pathways to provide cellular energy.

**Table 1.** SYBR primers (Sigma-Aldrich)

Atg13	KiCqStart primers M_Atg13_1
Atg4b	KiCqStart primers M_Atg4b_2
Gm2a	KiCqStart primers M_Gm2a_1
Hexb	KiCqStart primers M_Hexb_1
Lamp2	KiCqStart primers M_Lamp2_1
Gapdh	Fw: CCC​ACT​AAC​ATC​AAA​TGG​GG
	Rv: CCT​TCC​ACA​ATG​CCA​AAG​TT

**Table 2.** TaqMan probes (Thermo Fisher Scientific)

Nanog	Mm02384862_g1
Rex1	Mm03053975_g1
Elf5	Mm00468732_m1

## Discussion

Apart from the known direct targets of OCT4, such as *Utf1* (41), expression of most other pluripotency-associated genes, including the essential embryonic factors NANOG, SOX2, and ESRRB, is not significantly reduced in MUT cells compared with WT/HETs at the mid-blastocyst stage (E3.5) at both the mRNA and protein level (Fig. 1 and *SI Appendix*, Fig. S1). Detection of most pluripotency-associated factors in OCT4 MUT mid-blastocysts suggests independence from OCT4 at this stage, providing further evidence that the state of naive pluripotency, as captured in the form of ESCs in vitro, is not yet attained by the E3.5 ICM, as reported previously (76). Absence of *Utf1* expression implicates OCT4 indirectly in governing the epigenetic landscape of pluripotent cells, which may account for the precocious expression of some TE factors in E3.5 MUT cells, preceding changes in expression of most pluripotency genes. Surprisingly, *Cdx2*, previously implicated as a master repressor of *Pou5f1* in vitro (77), was not among the early-activated TE factors. This revelation highlights the caution with which behavior of ESCs can be extrapolated to the developing mammalian embryo. The possibility to perform detailed transcriptome analysis at the single-cell level has led to amendment of the previous assumption that loss of OCT4 in the embryo simply causes diversion to TE (13). The discovery that TE factors such as *Cdx2* and *Tead4* are poorly represented in mid-blastocyst ICMs following *Oct4* deletion provides evidence that this is not the case. However, the increase we observed in genes associated with lysosomes and autophagy factors as well as reduction in most KATs enzymes (Fig. 4) suggest that the response to the stress of loss of *Oct4* is largely metabolic. We used a recently developed auxin degron system that can induce relatively rapid depletion of OCT4 protein in ESCs (72) to substantiate the role of OCT4 in metabolic processes (Fig. 4*H* and *SI Appendix*, Fig. S4 *F* and *G*).

Another putative OCT4 target, *Il6st/gp130*, is a coreceptor essential for STAT3 signaling in ESCs (78). We observed significant down-regulation of STAT3 target genes in E3.5 MUT cells as well as reduced P-STAT3 protein and its pluripotency-associated targets TFCP2L1 and KLF4 (46, 47). Interestingly, diversion of ICM cells to TE has been observed in a proportion of embryos following maternal/zygotic deletion of *Stat3,* which was attributed to loss of activation of *Oct4* (44). Our study, however, implicates placement of OCT4 upstream of *Stat3*.

Signaling pathways related to matrix organization, including regulation of actin cytoskeleton and cell adhesion molecules, are significantly affected in E3.5 MUT cells. Such processes are associated with exit from pluripotency (79); cytoskeletal conformational changes inducing cell spreading are associated with differentiation. Our results therefore implicate OCT4 as a mediator for regulation of the biophysical properties of undifferentiated cells.

In this study, we dissected the role of metabolism in OCT4 MUT cells. We linked the reduction of glycolysis with the down-regulation of most *Kats* enzymes, which rely on acetyl-CoA, a product of glycolysis, to acetylate the lysine residues on histone proteins and maintain an open chromatin structure, associated with pluripotency. We revealed that most enzymes in glycolytic pathways are down-regulated in MUT cells. This may be because some rate-limiting enzymes (*Hk2*, *Pgk1*, *Pkm*, and *Eno1*) are potential targets of OCT4. We also noted down-regulation in MUT cells of genes associated with cell respiration. This is possibly a downstream effect of reduced STAT3 signaling, consistent with promotion of oxidative respiration via STAT3 for maintenance and induction of pluripotency (80). Consequently, respiration processes are disrupted in OCT4 MUT cells. Our scRNAseq data indicate that the lysosomal pathway is specifically activated in MUT cells as they transition toward TE. We propose that MUT cells up-regulate lysosomal gene expression and autophagy to counteract the down-regulation of glycolysis and the tricarboxylic acid cycle.

The requirement for OCT4 in development of the human embryo appears to be even more fundamental than for the mouse (81); OCT4 is apparently essential for formation of all three of the founder lineages in the human embryo. Consequently, no human embryo in which OCT4 was successfully deleted in all cells could advance beyond the 8-cell stage. Interestingly, absence of OCT4 in cells within mosaic embryos was consistently associated with loss of other pluripotency factors, contrasting with the published phenotype of OCT4 deletion in murine embryos (24, 25, 81). Furthermore, the presence of OCT4 null cells in mosaic embryos also exerted a detrimental effect upon nondeleted cells. The authors used a similar CRISPR-Cas9–mediated genome editing strategy for deletion of OCT4 in mouse embryos and recapitulated the previously published mouse phenotype, consistent with the results we present here.

**Fig. 5.**
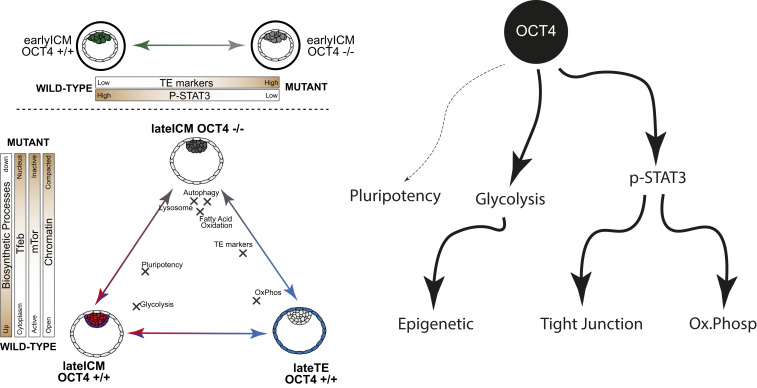
Scheme of OCT4 function in preimplantation embryo development.

In summary, our systematic analysis at the single-cell level in mouse embryos reveals an in vivo function for OCT4 in activating JAK/STAT signaling and regulating metabolic and biophysical cellular properties via energy metabolism, cell morphology, and chromatin accessibility for establishment of pluripotency in the developing mouse embryo (Fig. 5).

## Materials and Methods

Experiments were performed in accordance with European Union guidelines for the care and use of laboratory animals and under the authority of appropriate UK governmental legislation. The use of animals in this project was approved by the Animal Welfare and Ethical Review Body for the University of Cambridge, and relevant Home Office licenses are in place.

### Mice and Husbandry.

All embryos were generated from transgenic mouse strains with mixed genetic backgrounds. They were as follows: Oct4^+/−^ (13), ZP3CreTg^/+^ (82), R26::CreERT2 (83), and Oct4^LoxP/LoxP^ (25). Compound transgenic mice were generated from crosses of these lines. Genotyping was performed by PCR analysis using DNA extracted from ear biopsies or TE lysate following isolation of ICMs by immunosurgery (13, 27). Primer sequences are as follows: Oct4LoxP: CTC​AAA​CCC​CAG​GTG​ATC​TTC​AAA​AC and GGA​TCC​CAT​GCC​CTC​TTC​TGG​T; Oct4 null: GCC​TTC​CTC​TAT​AGG​TTG​GGC​TCC​AAC​C, GGG​CTG​ACC​GCT​TCC​TCG​TGC​TTT​ACG, and GAG​CTT​ATG​ATC​TGA​TGT​CCA​TCT​CTG​TGC; and Cre transgene: GCG​GTC​TGG​CAG​TAA​AAA​CTA​TC and GTG​AAA​CAG​CAT​TGC​TGT​CAC​TT. Amplification was carried out on around 5 µL lysate for 35 cycles (following 95 °C hot start for 10 min) of 94 °C for 15 s, 60 °C for 12 s, and 72 °C for 60 s with a final extension at 72 °C for 10 min. Reaction products were resolved by agarose gel electrophoresis. Mice were maintained on a lighting regime of 14:10 h light:dark with food and water supplied ad libitum. Embryos for RNA-seq were generated from Oct4^+/−^ inter se natural mating; those for IHC were compound transgenics derived from Oct4^LoxP/-^, ZP3Cre^Tg/+^ stud males, and Oct4^LoxP/LoxP^ dams. Detection of a copulation plug following natural mating indicated E0.5. Embryos were isolated in M2 medium (Sigma) at E3.5 or E4.5.

### Imaging.

Samples were observed using the Leica TCS SP5 confocal microscope. A 40× objective lens was used with Type F immersion liquid. Quantitative immunofluorescence was performed using modular interactive nuclear segmentation (MINS) to segment and quantify nuclei (volume, xyz-centroid, and fluorescence) on a per embryo basis (37). These data were fed into a MATLAB analysis pipelines. In brief, Delaunay triangulation was performed on the centroid of all nuclei to generate an in silico embryo surface. Next, the distance of each nuclear centroid to each face of the triangulated surface was calculated. The minimum distance and variance of distances to the surface was used to perform *k*-means clustering to prescribe an identity of either inside, ICM cells with larger minimum distance and lower variance, or outside, TE with smaller minimum distance and higher variance. Finally, the nonparametric Kruskal–Wallis test was performed to determine if the expression levels of cells with a tissue differ between genotypes.

### Preparation of Samples for RNA Sequencing.

For E3.5 blastocysts, zona pellucidae were removed using acid tyrode’s solution (Sigma) and embryos subjected to immunosurgery (13, 27) using 20% anti-mouse whole antiserum (Sigma) in N2B27 at 37 °C, 7% CO_2_ for 30 min, followed by three rinses in M2 and then 15 min in 20% nonheat inactivated rat serum (made in house) in N2B27 at 37 °C, 7% CO_2_. After 30 min in fresh N2B27, lysed TE was removed and placed in lysis buffer for genotyping. ICMs were incubated in 0.025% trypsin (Invitrogen) plus 1% chick serum (Sigma) for 5 to 10 min in small drops and dissociated by repetitive pipetting using a small diameter, mouth-controlled, flame-pulled Pasteur pipette. Individual ICM cells were transferred into single-cell lysis buffer and snap frozen on dry ice. Smart-seq2 libraries were prepared as described previously (84) and sequenced on the Illumina platform in a 125-bp paired-end format.

### RNA-Seq Data Processing.

Early/mid and late TE cells were downloaded from GSE45719. Genome build GRCm38/mm10 and STAR (spliced transcripts alignment to a reference) 2.5.2a (85) were used for aligning reads and Ensembl release 87 (86) was used to guide gene annotation. After removal of inadequate samples according to filtering criteria previously described (28), alignments were quantified to gene loci with htseq-count (87) based on annotation from Ensembl 87. Data are available under accession number GSE159030.

### Transcriptome Analysis.

Principal component and cluster analyses were performed based on log_2_FPKM values computed with custom scripts, in addition to the Bioconductor packages *DESeq* (88) or *FactoMineR*. Diffusion maps and t-distributed stochastic neighbor embedding were produced with *destiny* (89) and *Rtsne* packages. Diffusion map is a method for dimensionality reduction often used to analyze single-cell gene expression data, specifically to identify bifurcation and pseudotimes. Default parameters were used unless otherwise indicated. Differential expression analysis was performed with R package *scde* (90), which has the advantage of fitting individual error models for the assessment of differential expression between sample groups. For global analyses, we considered only genes with FPKM > 0 in at least one condition. Euclidean distance and average agglomeration methods were used for cluster analyses unless otherwise indicated. Expression data are made available in *SI Appendix*, Tables S1–S7 and through a web application to visualize transcription expression and fitted curve with temporal pseudotime of individual genes in embryonic lineages (https://giulianostirparo.shinyapps.io/pou5f1/). High variable genes across cells were computed according to the methods described (28, 40). A nonlinear regression curve was fitted between average log_2_FPKM and the square of coefficient of variation (logCV^2^); then, specific thresholds were applied along the *x*-axis (average log_2_FPKM) and *y*-axis (logCV^2^) to identify the most variable genes.

To assess the accuracy of the identified lineages, we used the WGCNA unsupervised clustering method (91) to identify specific modules of coexpressed genes in each developmental lineage/genotype. R package ggtern was used to compute and visualize ternary plots. KEGG was used to compute pathway enrichment and to download genes in glycolysis/gluconeogenesis and tricarboxylic acid cycle pathways.

### Quadratic Programming.

Fractional identity between preimplantation stages was computed using R package DeconRNASeq. (64). This package uses quadratic programming computation to estimate the proportion of distinctive types of tissue. The average expression of preimplantation stages (E4.5 WT/HET EPI and PrE, E4.5 MUT cells) was used as the “signature” dataset. Finally, the fraction of identity between TE cells and the “signature” dataset was computed using the overlapping gene expression data (FPKM > 0).

### ESCs and Culture.

Indole-3-acetic acid (IAA, Sigma) inducible Oct4 deletable pluripotent stem cells have recently been described (72). For TFEB staining, expanded colonies were passaged in standard N2B27 + 2iL. A total of 0.8 µg pPB-CAG-GFP-IRES Zeocin (gift from Masaki Kinoshita) and 0.4 µg pPy-CAG PBase were transfected to these cells using Lipofectamine 2000 (Thermo Fisher Scientific). The transfected cells were picked after selection with Zeocin (100 mg/mL), expanded, and routinely maintained on 0.1% gelatin-coated (Sigma) 6-well plates (Falcon) in N2B27 + 2iL. They were passaged every 3 d following dissociation with Accutase.

#### Cell differentiation.

IAA inducibly depletable OCT4 cells were seeded (1.5 × 10^4^) on fibronectin-coated (12.5 µg/mL; Millipore) ibidi dishes (µ-Dish, 35 mm) and cultured in N2B27 + 2iL for 1 d. The next day, the medium was switched to N2B27 + 100 U/mL LIF (leukemia inhibitory factor), 3 µM CHIR (Chiron 99021), and 500 µM IAA for OCT4 deletion (or 0.1% ethanol for controls), and cells were cultured for another day before analysis was performed.

### IHC.

Embryos were immunostained as described previously (25). Primary antibodies used in the present study are listed in *SI Appendix*, Table S6.

OCT4-deleted and control ESCs were fixed with 4% paraformaldehyde in phosphate-buffered saline (PBS) at room temperature for 15 min, then rinsed in PBS and blocked in PBS containing 3% donkey serum (Sigma) and 0.1% Triton X at 4 °C for 2 to 3 h. Primary antibodies (*SI Appendix*, Table S7) were diluted in blocking buffer, and samples were incubated in the appropriate antibody solution at 4 °C overnight. They were rinsed three times in PBST, comprising PBS + 0.1% Triton X, for 15 min each. Secondary antibodies were diluted in blocking buffer with or without 500 ng/mL DAPI, and samples were incubated in the appropriate secondary antibody solution at room temperature for 1 h in the dark. They were rinsed three times in PBST for 15 min each then stored in PBS at 4 °C in the dark until imaging.

### Western Blot.

For Western blotting, tris-buffered saline (TBS)–Tween buffer (pH = 7.4) was made as follows: 137 mM NaCl, 2.7 mM KCl, 0.25 mM Trizma solution, 1 mL Tween-20 (all Sigma), and deionized water to the final volume of 1 L. For p-STAT3 Western, membrane blocking was performed for 24 h in TBS-Tween + 5% bovine serum albumin (BSA) at 4 °C followed by 16 h incubation in 1:1,000 monoclonal anti-Y705pSTAT3 rabbit primary antibody (catalog number 9145; Cell Signaling Technology) in TBS–Tween + BSA at 4 °C. The membrane was washed three times in TBS–Tween and incubated with 1:10000 horseradish peroxidase (HRP)-linked anti-rabbit IgG secondary antibody (catalog number NA934V; GE Healthcare) for 1 h and then washed three times in TBS–Tween and incubated with Enhanced Chemiluminescent Reagent (Amersham). Detection was performed on an X-ray film (Fujifilm).

For tubulin Western, the membrane was blocked for 1 h, incubated with 1:2,000 monoclonal anti–α-tubulin mouse (catalog number 7291; Abcam) for 30 min, and washed three times in TBS–Tween buffer. Then, the membrane was blocked again for 1 h and incubated with 1:10000 HRP-linked anti-mouse IgG secondary antibody (catalog number NA931V; GE Healthcare) for 1 h, followed by the same procedures as described for pSTAT3 western.

### qRT-PCR.

Total RNA was isolated using RNeasy Mini Kit (Qiagen) and DNase treatment (Qiagen). Specifically, 500 ng RNA was reverse-transcribed with SuperScript III First-Strand Synthesis SuperMix for qRT-PCR (Thermo Fisher Scientific), and the obtained complementary DNA was analyzed by qRT-PCR using TaqMan Fast Universal PCR Master Mix (Thermo Fisher Scientific) or Fast SYBR Green Master Mix (Thermo Fisher Scientific). Reactions were performed in triplicates in 96-well or 384-well plates (Thermo Fisher Scientific) and analyzed using StepOnePlus Real-Time PCR System (Applied Biosystems) or QuantStudio 12K Flex system (Applied Biosystems). Gene expression was normalized to *Gapdh* and reference samples indicated specifically. The TaqMan assay identification is Mm00658129_gH.

### Plasmids.

*PB.TetO.Oct4.PGK.hph* is a PiggyBac plasmid that enables *Oct4* expression under doxycycline inducible promoter/operator (Tet-On system) and constitutive expression of hygromycin B resistance marker (hygromycin B phosphotransferase, hph).

*PB.CAG.rtTA3.PGK.pac* is a PiggyBac plasmid that enables constitutive rtTA expression coupled with puromycin resistance marker (Puromycin N-acetyltransferase, pac).

*CAG.PBase* encodes a constitutively expressed PBase to enable chromosome integration of PiggyPac plasmids.

To design a dox-inducible *Oct4* ESC line, E14tg2a cells (500,000 cells per well with a 6-well plate) were cotransfected with 1 µg *PB.CAG.rtTA3.PGK.pac* and 0.5 µg *CAG.PBase* using Lipofectamine 2000 (Invitrogen) in a total of 2 mL culture medium. Transfection medium was withdrawn, and a fresh culture medium was applied 8 h post-transfection. Transfectants were selected for a month on 50 to 150 μg/mL hygromycin B (Life Technologies) combined with 0.33 to 1.00 µg/mL puromycin (Thermo Fisher).

## Supplementary Material

Supplementary File

Supplementary File

Supplementary File

Supplementary File

Supplementary File

Supplementary File

Supplementary File

Supplementary File

## Data Availability

RNA sequencing data have been deposited in Gene Expression Omnibus (GSE159030). All other study data are included in the article and supporting information.
