# Supplementary material for: OCT4 induces embryonic pluripotency via STAT3 signaling and metabolic mechanisms
Source: Proc Natl Acad Sci U S A. 2021 Jan 15;118(3):e2008890118. doi: 10.1073/pnas.2008890118 (PMC7826362; doi:10.1073/pnas.2008890118)
Supplement: Supplementary File [file pnas.2008890118.sapp.pdf]

PNAS
www.pnas.org

Supplementary Information for

Oct4 induces embryonic pluripotency via Stat3 signalling and metabolic mechanisms

Giuliano G. Stirparo, Agata Kurowski, Ayaka Yanagida, Lawrence E Bates, Stanley E. Strawbridge, Siarhei Hladkou, Hannah Stuart, Thorsten E. Boroviak, Jose C. R. Silva and Jennifer Nichols

Email: jn270@cam.ac.uk

This PDF file includes:

Supplementary text
Figures S1 to S4
Tables S1 to S7

Supplementary Information Text

Fig. S1. (A) t-SNE plot for early blastocyst cells. Sample colors represent Pou5f1 log2 expression. (B) Heatmap showing the top co-regulated genes between E3.5 MUT and E3.5 WT/HET (> 5 interaction; n=346 MUT genes and n=398 WT/HET genes). (C) Boxplots for Nanog, Klf4 and Esrrb expression. (D) FPKM expression of genes associated with pluripotency and mean ± sd for WT/HET and MUT cells (Padj Nanog:0.3, Sox2:0.74, Esrrb:0.75, Utf1:2.32e-10). (E) Confocal images and normalized expression of OCT4 HET and MUT embryos stained for SOX2 and ESRRB (dotted lines represent ICM). (F) FPKM expression of Stat3 in WT, HET and MUT cells. (G) PCA computed with genes in KEGG JAK/STAT signalling pathway. Each dot represents a cell. (H) Cumulative sum of relative percentages between the expression of genes in STAT3 pathway (KEGG); student's t-test p.val < 0.05. (I) Total Oct4 level in iOct4 ESCs (J) Western blot for pSTAT3 after induction of Oct4.

Fig. S2. (A) Diffusion plot of early and late blastocyst cells; color represents Pou5f1 expression. (B) Barplot shows FPKM expression of Pou5f1 for each single cell at E4.5 stage. (C) One-way hierarchical cluster of eigengenes value computed from WGCNA (power 8; dist=0.35, size =30). (D) Density distribution of log2FPKM in late blastocyst PrE and MUT cells of 667 genes and late blastocyst EPI and MUT cells of 517 genes. (E) Scatter plot of Nanog and Gata6 FPKM expression values for E4.5 EPI (WT/HET), E4.5 PrE (WT/HET) and E4.5 MUT cells. (F) log2FPKM expression of Fgf4, Fgfr1 and Fgfr2 in WT/HET EPI/PrE and MUT cells. (G) log2FPKM scatter plot of MAPK signalling genes between late PrE and MUT blastocyst cells. (H)Ternary plot of early WT/HET blastocyst cells, WT/HET EPI and TE cells. Axes show the density of the relative fraction of expression.

Fig. S3. (A) GSEA analysis computed with log2FC between E4.5 EPI-WT.HET/E4.0 TE and (B) E4.5 MUT/E4.0 TE. (C) Distribution of expression values in WT and MUT Oct4 ESC for Fabp3, Cldn4 and (D) Lats2 and Amotl2. (E) Barplot of OCT4 CHIP targets in ESC. (F) Pathways enriched for the top1000 OCT4 CHIP targets in ESC.

Fig. S4. (A)TCA associated enzymes (arrows) colored by the ratio between E4.5 WT and MUT cells and heatmap of the associated enzymes. (B) Volcano plot showing the contribution on principal component 1 (Fig.4A) and OCT4 ChIP-seq score in ESCs. (C) Venn diagram of KEGG pathways enriched for variable genes in E4.5 EPI/PrE vs MUT and between OCT4 depleted ESC vs control. (D) Scatter plot of autophagy related genes (Atgs) between E4.5 WT/HET ICM and E4.5 MUT. (E) Enzymes in fatty acid oxidation and synthetase; color represents the ratio between E4.5 WT/HET and MUT cells. (F) RT-qPCR analysis of gene expression in a time-course following OCT4 degradation in ESCs (days following addition of IAA in 2iL), normalized to the highest value for each gene. Bars represent the mean of three technical replicates, error bars indicate standard deviation. (G) TFEB localization in OCT4+/+ and OCT4-/- ESCs cultured in CHIR+LIF

Supplementary Tables

Table S1. Table_S1. Number of cells and embryo of origin used in this study.

Table S2. Table_S2. Genes co-regulated identified with WGCNA

Table S3. Table_S3. Average score of OCT4 CHIP target in ESC.

Table S4. Table_S4. Genes co-regulated identified with WGCNA

Table S5. Table_S5. Average gene expression table containing all samples analyzed in this study.

Table S6. Table_S6. Scheme of the antibodies used for IF.

Table S7. Table_S7. Significance and mean fluorescence for IF data.

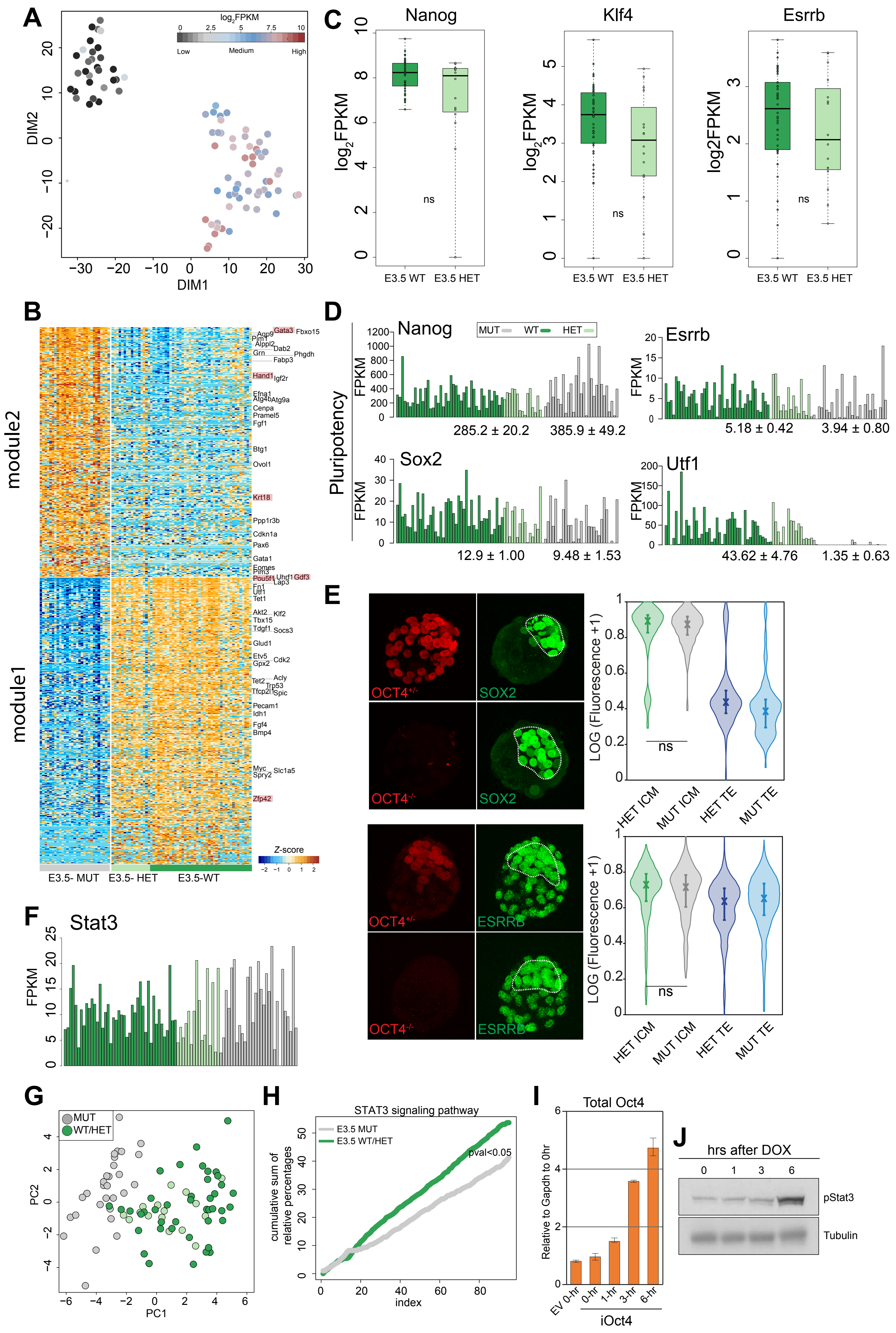
A
C
Nanog
Klf4
Esrrb
log_2FPKM
DIM1
DIM2
Low
Medium
High
ns
E3.5 WT
E3.5 HET
log2FPKM
B
D
module2
module1
Gata3
Hand1
Krt18
Pou5f1
Zfp42
Z-score
E3.5- MUT
E3.5- HET
E3.5-WT
Pluripotency
FPKM
MUT
WT
HET
Sox2
Utf1
285.2 ± 20.2
385.9 ± 49.2
5.18 ± 0.42
3.94 ± 0.80
12.9 ± 1.00
9.48 ± 1.53
43.62 ± 4.76
1.35 ± 0.63
E
OCT4+/-
OCT4-/-
SOX2
ESRRB
LOG (Fluorescence +1)
HET ICM
MUT ICM
HET TE
MUT TE
F
Stat3
G
MUT
WT/HET
PC1
PC2
H
STAT3 signaling pathway
E3.5 MUT
E3.5 WT/HET
pval<0.05
cumulative sum of relative percentages
index
I
Total Oct4
Relative to Gapdh to 0hr
EV 0-hr
0-hr
1-hr
3-hr
6-hr
iOct4
J
hrs after DOX
pStat3
Tubulin

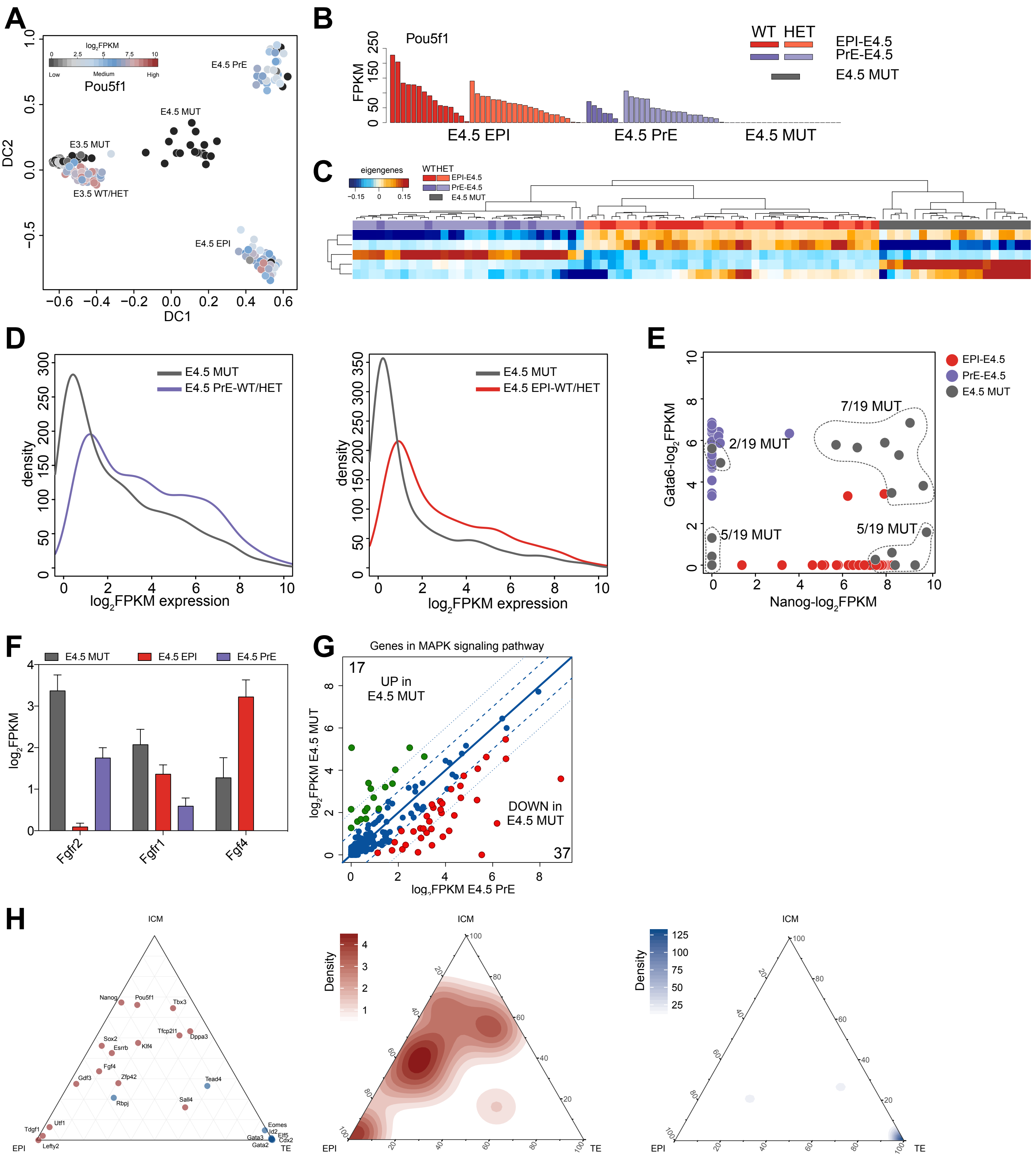

A
log2FPKM
Low
Medium
High
Pou5f1
E4.5 PrE
E4.5 MUT
E3.5 MUT
E3.5 WT/HET
E4.5 EPI
DC1
DC2
B
Pou5f1
WT HET
EPI-E4.5
PrE-E4.5
E4.5 MUT
FPKM
E4.5 EPI
E4.5 PrE
E4.5 MUT
C
eigengenes
WTHET
EPI-E4.5
PrE-E4.5
E4.5 MUT
D
E4.5 MUT
E4.5 PrE-WT/HET
density
log2FPKM expression
E4.5 MUT
E4.5 EPI-WT/HET
density
log2FPKM expression
E
EPI-E4.5
PrE-E4.5
E4.5 MUT
7/19 MUT
2/19 MUT
5/19 MUT
5/19 MUT
Gata6-log2FPKM
Nanog-log2FPKM
F
E4.5 MUT
E4.5 EPI
E4.5 PrE
log2FPKM
Fgfr2
Fgfr1
Fgf4
G
Genes in MAPK signaling pathway
17
UP in
E4.5 MUT
DOWN in
E4.5 MUT
37
log2FPKM E4.5 MUT
log2FPKM E4.5 PrE
H
ICM
EPI
TE
Nanog
Pou5f1
Tbx3
Tfcp2l1
Dppa3
Sox2
Esrrb
Klf4
Fgf4
Gdf3
Zfp42
Tead4
Rbpj
Sall4
Tdgf1
Utf1
Lefty2
Eomes
Id2
Elf5
Cdx2
Gata3
Gata2
Density

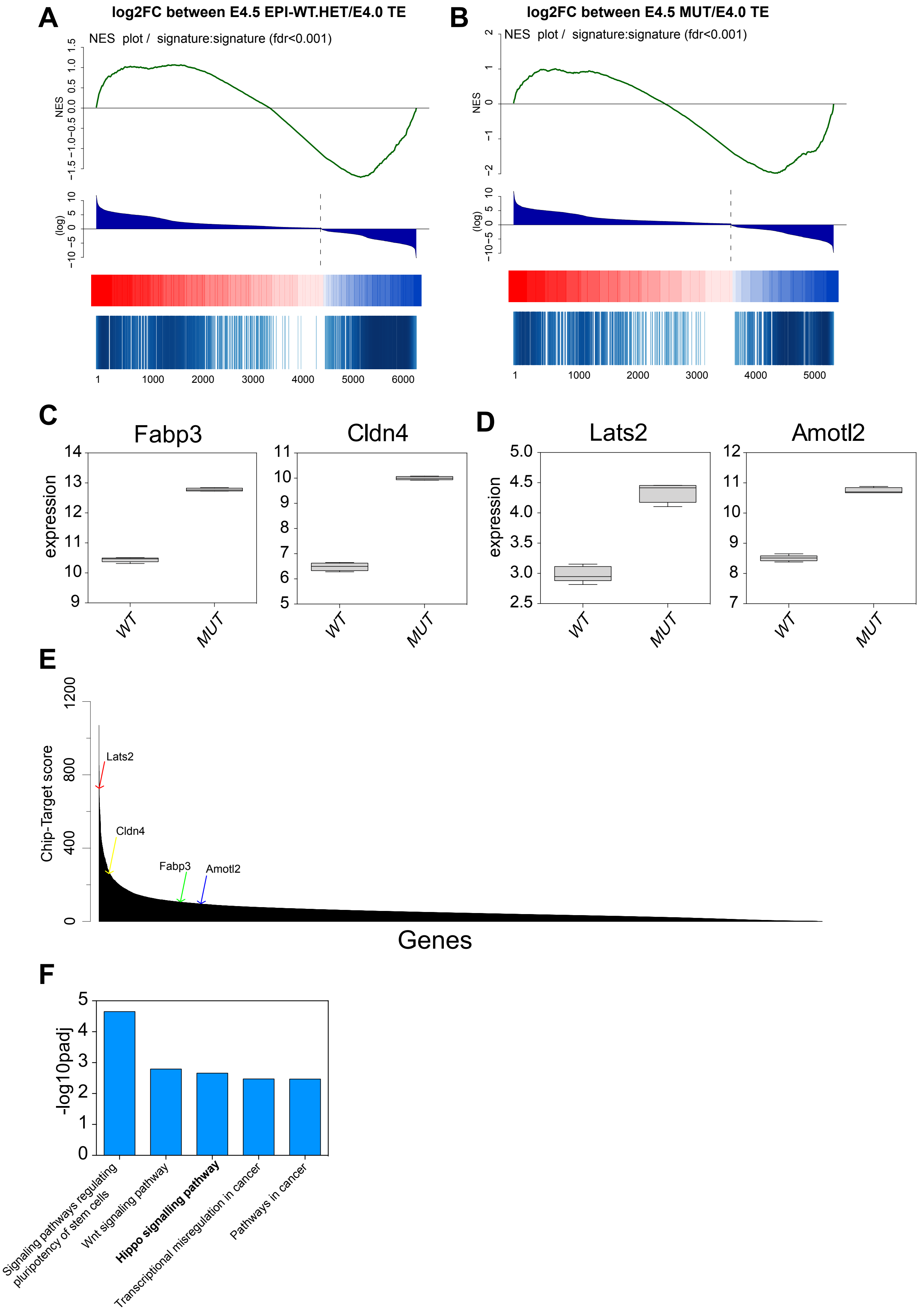
A
log2FC between E4.5 EPI-WT.HET/E4.0 TE
NES plot / signature:signature (fdr<0.001)
NES
(log)
B
log2FC between E4.5 MUT/E4.0 TE
NES plot / signature:signature (fdr<0.001)
C
Fabp3
Cldn4
expression
WT
MUT
D
Lats2
Amotl2
E
Chip-Target score
Lats2
Cldn4
Fabp3
Amotl2
Genes
F
-log10padj
Signaling pathways regulating pluripotency of stem cells
Wnt signaling pathway
Hippo signalling pathway
Transcriptional misregulation in cancer
Pathways in cancer

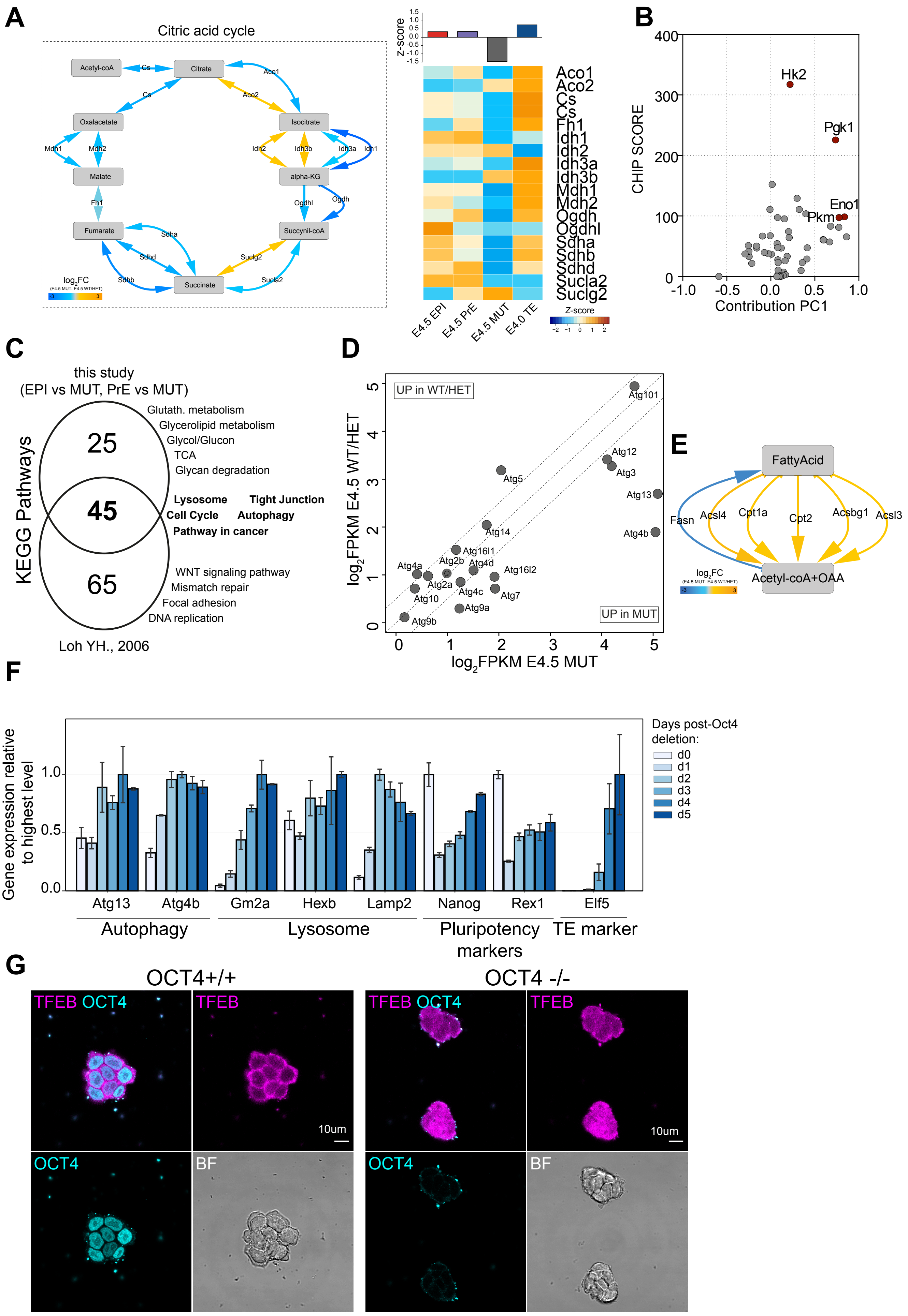

A
Citric acid cycle
Acetyl-coA
Citrate
Oxalacetate
Isocitrate
Malate
alpha-KG
Fumarate
Succynil-coA
Succinate
Cs
Aco1
Aco2
Mdh1
Mdh2
Idh2
Idh3b
Idh3a
Idh1
Fh1
Ogdhl
Ogdh
Sdha
Sdhd
Sdhb
Sucl g2
Sucla2
log2FC
(E4.5 MUT- E4.5 WT/HET)
Z-score
Aco1
Aco2
Cs
Cs
Fh1
Idh1
Idh2
Idh3a
Idh3b
Mdh1
Mdh2
Ogdh
Ogdhl
Sdha
Sdhb
Sdhd
Sucla2
Suclg2
E4.5 EPI
E4.5 PrE
E4.5 MUT
E4.0 TE
Z-score
B
CHIP SCORE
Contribution PC1
Hk2
Pgk1
Eno1
Pkm
C
this study
(EPI vs MUT, PrE vs MUT)
KEGG Pathways
25
45
65
Glutath. metabolism
Glycerolipid metabolism
Glycol/Glucon
TCA
Glycan degradation
Lysosome
Tight Junction
Cell Cycle
Autophagy
Pathway in cancer
WNT signaling pathway
Mismatch repair
Focal adhesion
DNA replication
Loh YH., 2006
D
UP in WT/HET
UP in MUT
log2FPKM E4.5 WT/HET
log2FPKM E4.5 MUT
Atg101
Atg12
Atg3
Atg13
Atg4b
Atg5
Atg14
Atg16l1
Atg4a
Atg2b
Atg4d
Atg16l2
Atg2a
Atg4c
Atg10
Atg7
Atg9a
Atg9b
E
FattyAcid
Acetyl-coA+OAA
Fasn
Acsl4
Cpt1a
Cpt2
Acsbg1
Acsl3
log2FC
(E4.5 MUT- E4.5 WT/HET)
F
Gene expression relative to highest level
Days post-Oct4 deletion:
d0
d1
d2
d3
d4
d5
Atg13
Atg4b
Gm2a
Hexb
Lamp2
Nanog
Rex1
Elf5
Autophagy
Lysosome
Pluripotency markers
TE marker
G
OCT4+/+
OCT4 -/-
TFEB OCT4
TFEB
OCT4
BF
10um
